# How do we use therapeutic drug monitoring to improve outcomes from severe infections in critically ill patients?

**DOI:** 10.1186/1471-2334-14-288

**Published:** 2014-11-28

**Authors:** Gloria Wong, Fekade Bruck Sime, Jeffrey Lipman, Jason A Roberts

**Affiliations:** Burns Trauma and Critical Care Research Centre, The University of Queensland, Brisbane, Queensland Australia; School of Pharmacy and Medical Sciences, University of South Australia, Adelaide, Australia; Therapeutics Research Centre, Basil Hetzel Institute for Translational Health Research, The Queen Elizabeth Hospital, Adelaide, Australia; Royal Brisbane and Women’s Hospital, Level 3 Ned Hanlon Building, Butterfield St, Brisbane, 4029 Queensland Australia

**Keywords:** TDM, Antibiotic, Pharmacokinetics, Pharmacodynamics

## Abstract

High mortality and morbidity rates associated with severe infections in the critically ill continue to be a significant issue for the healthcare system. In view of the diverse and unique pharmacokinetic profile of drugs in this patient population, there is increasing use of therapeutic drug monitoring (TDM) in attempt to optimize the exposure of antibiotics, improve clinical outcome and minimize the emergence of antibiotic resistance. Despite this, a beneficial clinical outcome for TDM of antibiotics has only been demonstrated for aminoglycosides in a general hospital patient population. Clinical outcome studies for other antibiotics remain elusive. Further, there is significant variability among institutions with respect to the practice of TDM including the selection of patients, sampling time for concentration monitoring, methodologies of antibiotic assay, selection of PK/PD targets as well as dose optimisation strategies. The aim of this paper is to review the available evidence relating to practices of antibiotic TDM, and describe how TDM can be applied to potentially improve outcomes from severe infections in the critically ill.

## Review

### Introduction

Despite advances in contemporary medicine, severe infections and sepsis-related mortality in critically ill patients remain a global problem [1–4]. An important consideration of antimicrobial treatment failure in the critically ill is inadequate drug exposure from use of dosing regimens derived in non-critically ill patients [5]. There is extensive evidence of sub-therapeutic exposure from standard doses across different antibiotic classes including beta-lactams [6, 7], aminoglycosides [8], glycopeptides [9], fluoroquinolones [10], and oxazolidinones [11]. This can be a direct consequence of pharmacokinetic alterations emanating from the complex pathophysiologic processes associated with severe infection. Multi-drug resistant organisms more frequently encountered in the critically ill also alter the dosing requirements for these patients [12–14]. Appropriate, timely antibiotic therapy given at an adequate dose is thought to be of paramount importance in improving clinical outcome of severe sepsis [15]. To further increase the likelihood of achieving a good patient outcome from infection, optimizing antibiotic dosing is crucial. Therapeutic drug monitoring (TDM), a tool traditionally used primarily to minimize toxicity in drugs with narrow therapeutic window or drugs with complex pharmacokinetics, is being increasingly used for antibiotic dose optimization in the attempt to improve attainment of pharmacokinetic/pharmacodynamic (PK/PD) targets and outcomes of severe infections in the critically ill [16–19].

Despite a growth in practice of antibiotic TDM globally, clinical outcome studies on TDM-based interventions are limited. To date, definitive benefit is only demonstrated for aminoglycosides [20, 21]. Further, there is significant variability among institutions with respect to the practice of TDM including the selection of patients, sampling time for concentration monitoring, methodologies of antibiotic assay, selection of PK/PD target as well as dose optimisation strategies [22]. The aim of this paper is therefore to critically review the available evidence of the practices of antibiotic TDM, and describe how TDM can be utilised to potentially improve critically ill patient outcomes from severe infections.

### Pharmacokinetic changes in the critically ill

Altered pharmacokinetics of antibiotics in ICU patients with severe infections secondary to dynamic disease processes and medical interventions has been widely described [10, 11, 23, 24]. Altered drug exposure may also be observed in patients with severe sepsis outside the ICU. Specifically, changes in volume of distribution (V_d_) and drug clearance (Cl) may lead to sub-therapeutic or toxic exposures of antibiotics when standard doses are used. Table 1 describes common factors that may alter pharmacokinetics of antibiotics in critically ill patients. Fluid resuscitation, capillary leakage and third space losses could substantially increase V_d_ of hydrophilic antibiotics such as beta-lactams and aminoglycosides, the V_d_ of which approximates the extracellular fluid volume. The Cl of antibiotics is dependent on patient organ function, drug clearance mechanisms and extracorporeal interventions given to the patient. Renal hypoperfusion, acute kidney injury and end-organ failure decrease Cl of antibiotics. On the contrary, augmented renal clearance (ARC) has been described in critically ill patients, where increased elimination of antibiotics lead to sub-therapeutic concentrations [25, 26]. The impact of interventions such as renal replacement therapy (RRT) and extracorporeal membrane oxygenation (ECMO) on antibiotic pharmacokinetics is multifactorial, variable and complex and have been discussed in detail elsewhere [27–30].

**Table 1:** **Summary of common factors associated with altered pharmacokinetics of antibiotics in critically ill patients**

Increased V_d_	Decreased Cl	Increased Cl	Variable changes in V_d_and/or Cl
Hypoalbuminaemia, leading to increased unbound drug	Renal hypoperfusion	Augmented renal clearance	Extracorporeal interventions (eg RRT, ECMO)
Capillary leakage	Acute kidney injury		
Fluid resuscitation	Renal/hepatic dysfunction		
Third space loss			

### Main pharmacokinetic/pharmacodynamic indices associated with antibiotic efficacy

The three main PK/PD indices that describe antibiotic exposure required for bacterial stasis or killing are summarised in Tables 2 and 3. The PK/PD targets for individual groups of antibiotics proven or proposed to be associated with clinical advantage based on animal and clinical studies are also listed. Of note, all PK/PD targets are expressed in relation to the minimal inhibitory concentration (MIC) of the pathogen, highlighting that beyond measurement of antibiotics concentrations, accurate and timely determination of MIC also should be considered a cornerstone of antibiotic TDM. MICs for TDM can be defined by various strategies, including Etest, disc method, micro-dilution broth method and automated microbiology system (e.g. Phoenix, Vitek 2), or adoption of local antibiograms, EUCAST and CLSI breakpoints. Clinicians utilising TDM in treating severe infections, especially those that involve resistant organisms, need to be aware of the limitations of each method. These limitations have been discussed in detail elsewhere [31–35].

**Table 2:** **Summary of time-dependent antibiotics and proposed targets for TDM dose adjustments**

PK/PD index	Antibiotics	PK/PD thresholds associated with optimal bacterial killing and/or clinical outcome	PK/PD threshold for potential toxicity
*f*T_>MIC_	Beta-lactams	Predominantly 100% *f*T_>MIC_ for TDM purposes [22]	Has not been clearly defined. Thresholds from 100% *f*T_>6xMIC_ to 100% *f*T_>10xMIC_ has been arbitrarily chosen by some centres [22]
	Penicillins	30% *f*T_>MIC_ (bacteriostatic)	
		50% *f*T_>MIC_ (bactericidal – animal model and clinical studies in non-critically ill patients) [154]	
	Cephalosporins	40–70% *f*T_>MIC_ (animal models) [154]	
		100% *f*T_>MIC_ up to 100% *f*T_>4–5xMIC_ (optimal clinical outcome observed for cefepime and ceftazidime) [18, 37]	
	Carbapenems	20% *f*T_>MIC_ (bacteriostatic)	
		40% *f*T_>MIC_ (bactericidal)	
		100% *f*T_>5xMIC_ (optimal clinical outcome observed for meropenem) [155]	
	Monobactams	50% *f*T_>MIC_ (bactericidal)	
	Linezolid	>85% *f*T_>MIC_[125, 156]

**Table 3:** **Summary of concentration-dependent with time-dependence and concentration-dependent antibiotics, and proposed targets for TDM dose adjustments**

	PK/PD indices	Antibiotics	PK/PD thresholds associated with optimal bacterial killing and/or clinical outcome	PK/PD threshold for potential toxicity
Concentration-dependent with time-dependence	AUC_0–24_/MIC (AUIC)	Vancomycin	a) AUIC ≥ 400 (corresponds to trough concentrations of 15–20 mg/Lfor intermittent dosing; trough of 20–25 mg/L for continuous dosing)	Trough concentrations >27 mg/L with intermittent dosing
			b) trough concentrations >10 mg/L to avoid development of resistance [16]	
		Linezolid	AUIC > 80 to 120 (corresponds to trough concentrations > 2 mg/L) [125, 156]	Has not been clearly defined
				Theoretical maximum trough concentrations threshold: 7–10 mg/L [120, 157, 158]
				Recommended maximum: 7 mg/L [158]
		Fluoroquinolones	AUIC > 125 for Gram negative organisms [137, 159, 160]	
		Aminoglycosides	Relation to therapeutic efficacy mainly shown in animal infection models	
		Daptomycin	AUIC > 666 [151]	Trough concentrations >24.3 mg/L [150]
Concentration-dependent	Peak (C_max_)/MIC	Fluoroquinolones	C_max_/MIC >10 prevent emergent of resistant mutants in *in vivo* and *in vitro* models [137, 161, 162]	
		Aminoglycosides	C_max_/MIC 8–10 [163]	High dose extended-dosing: troughs undetectable or <1 mcg/mL
		Daptomycin	C_max_/MIC 59–94 [152]	

*Abbreviations:* *f*T_*>MIC*_ percentage/fraction of dosing interval during which unbound antibiotic concentration remain above the MIC of targeted bacteria, *AUC*_*0–24*_ */MIC* ratio of the area under the concentration–time curve (AUC) of the unbound drug from 0–24 hour and the MIC of targeted bacteria, *Peak (C*_*max*_ *)/MIC* ratio of the peak concentration during a dosing interval and the MIC of targeted bacteria.

### How TDM could be utilised to optimize PK/PD in treating severe infections – clinical evidence and practical issues

#### Beta-lactams

Given the excellent safety profile of beta-lactams, the main aim of TDM with these antibiotics is to maximise efficacy through achievement of therapeutic exposures [36]. Targets required to achieve a favourable clinical outcome in the critically ill have been described to be higher than that supported by studies in animal models or in non-ICU patients (Table 2). A PK/PD target of 100% *f*T_>MIC_ as against *f*T_>MIC_ lower than 100% was associated with significantly greater clinical cure and bacteriological eradication in septic critically ill patients with bacteremia, lower respiratory tract or complicated urinary tract infection treated with cefepime and ceftazidime [18]. Tam *et al.* found similar associations against gram-negative infections, although proposed an even higher PK/PD target with cefepime (on concurrent aminoglycosides) [37]. Unfortunately, achievement of these higher drug concentrations in ICU patients is infrequent, especially in the early phase of sepsis [6, 38]. Other dosing strategies have been proposed for optimizing beta-lactams exposure, these include dose adjustments made specific to interventions, continuous infusion and dosing monograms. However, individual reports still demonstrate the extreme difficulties in achieving appropriate drug concentrations in some severely ill patients [30, 39–42]. TDM appeared to be a feasible strategy to adapt beta-lactam dosing and may complement these other measures [12, 43]. The potential benefit of beta-lactam TDM probably warrants evaluation of its value for treating severe infections in the critically ill, despite the fact that the optimal PK/PD target remains unclear.

Beta-lactams have a low likelihood of toxicity. However, given the high drug concentration requirement in some severely ill patients for difficult to treat infections, toxicity becomes an issue that could be minimize with TDM. No threshold of toxicity has been defined currently, however TDM could aid early recognition of potential drug-related toxicities (especially neurological toxicity) in susceptible patients [44–46].

##### Selection of patients

Patients with sepsis or septic shock, who potentially would benefit from TDM, are those with labile blood pressure (ie septic shock), dynamic renal function, burns injury, receiving RRT or ECMO, infected with resistant organisms, and where neurological toxicity is clinically suspected [22, 47, 48].

##### Sampling time

Trough concentrations sampled at steady state (generally after 3–4 doses given) are appropriate for determining whether PK/PD targets have been achieved. Additional sampling (e.g. at mid-dosing interval) is appropriate for a more accurate calculation of pharmacokinetic parameters and would be useful for a Bayesian-driven dose adaptation using appropriate computer software.

##### Assay

Liquid chromatography is the most widely used assaying method for beta-lactam TDM [49–53]. A variety of published protocols are available to suit clinical and laboratory needs in different institutes [22]. The high equipment and personnel costs as well as the relatively prolonged processing time (between 6–24 hours) are disadvantages of the method that might hinder the wider application of beta-lactam TDM. Direct measurement of unbound drug concentrations is also recommended in critically ill patients with hypoalbuminaemia receiving highly protein-bound antibiotics [54].

##### Dose adjustment strategies

Generalized but non-specific dose adjustment methods including changing dose amount or frequency, utilization of extended or continuous infusion have been used in most units practising TDM routinely [22]. New dosages can be determined more accurately by calculating the individual patient’s drug clearance from measured beta-lactam concentrations. Dosing nomograms and PK softwares for dose adjustment are available but have not been widely tested nor validated [39, 55].

#### Aminoglycosides

With activity against gram-negative bacteria, aminoglycosides are an inexpensive group of antibiotics frequently used in the ICU for treatment of severe infections. Once-daily administration to maximize its concentration-dependent effect and post-antibiotic effect is widely accepted as the standard regimen in general ward patients, and in ICU patients as well. For gentamicin, the regimen itself has been proven to provide small improvement in efficacy and/or reduced nephrotoxicity, and the benefit is augmented with active TDM [19, 56–59]. Gentamicin, tobramycin and amikacin are the three antibiotics mostly subjected to TDM. In non-critically ill patients, the aim of TDM for extended interval aminoglycoside dosing is mainly to reduce toxicity, as arguably the single high dose would provide an adequate C_max_ (maximum concentration in dosing interval) in most cases [60]. However, these doses in critically ill patients are associated with a decreased rate of achievement of peak and AUC (area under the concentration-time curve) targets [8, 61–64]. Although minimizing the likelihood of toxicity using TDM is important in critically ill patients, dose adaptation to avoid under-dosing and maximize efficacy is also valuable. Given the high mortality rate of severe infections in the critically ill, high variability in aminoglycoside PK, and the proven benefit of aminoglycoside TDM in general patient populations, TDM practice tailored to the critically ill population is advised.

##### Selection of patients

Measurement of C_max_ concentrations is advisable in patients with conditions associated with an increased V_d_ (eg. burns, septic shock). Those with unstable hemodynamic and/or renal function, undergoing RRT, infected with resistant pathogens would also benefit from routine TDM. AUC based monitoring is preferred, but where not possible, trough concentration monitoring to minimize toxicity is suggested especially for patients receiving regimens exceeding 48 hours [65–68].

##### Sampling time

Traditionally, measuring one random concentration between 6–14 hours after commencement of antibiotic infusion with interpretation using a nomogram has been used for aminoglycosides. Given these nomograms are more commonly developed in non-critically ill patients, use of this approach in the critically ill is not recommended [57, 69]. TDM with two samples drawn at 1 (30-mins post completion of drug infusion) and 6–22 hours post administration [70, 71] allows description of peak concentrations and AUC using linear regression or Bayesian approaches and a more accurate prediction of future dosing requirements.

##### Assay

Commercially available immunoassays are the most frequently used method for aminoglycoside TDM. Although other methods such as capillary zone electrophoresis and chromatography offer higher precision, the inexpensive immunoassays have been validated and are appropriate for routine daily clinical practice [72, 73].

##### Dose adjustment strategies

The PK/PD targets conventionally used for aminoglycoside TDM are described in Table 3. To the best of our knowledge, no other targets have been established especially for the critically ill population. Calculation of the AUC for an individual patient and subsequent dosage adjustment using dosing software should be considered the ideal approach. Although clinical advantages of using software based dosing methods have not been demonstrated, they should be considered preferred for critically ill patients with severe infections [74–76].

#### Vancomycin

The benefit of vancomycin TDM both for avoidance of toxicity as well as improving clinical outcome remains controversial. Conflicting evidence exists in regards to correlation of nephrotoxicity with high serum vancomycin concentrations [77–84]. A recent meta-analysis [85] concluded that the collective literature favours the association. However it is still debatable whether the high concentration or kidney damage is the preceding event. Similarly controversy exists with respect to ototoxicity [86, 87] as well as benefit in clinical outcome [17, 84, 88–90]. A meta-analysis by Ye *et al.*[91] suggested TDM significantly increases the likelihood of clinical efficacy and decreases the rate of nephrotoxicity. There is also a good agreement in the benefit of TDM to prevent the emergence of vancomycin resistant organisms with trough concentration above 10 mg/L [90, 92, 93].

##### Selection of patients

TDM is warranted to avoid toxicity in patients receiving high doses; during concomitant therapy with other nephrotoxic or ototoxic agents, in patients with unstable renal function, those receiving prolonged therapy (>3 to 5 days), during RRT and in hemodynamically unstable critically ill septic patients [16, 94].

##### Sampling time

Trough concentrations are modestly correlated with AUC enabling prediction of the target AUC/MIC [95, 96]. Based on available techniques, samples should be taken at pharmacokinetic steady state, which would usually be after about four doses (assuming 12-hourly dosing) [97]. In patients with renal dysfunction where half life is prolonged, steady state may not be achieved at the fourth dosing and therefore a trough concentration at this time may underestimate steady-state antibiotic exposure [97, 98]. This should be taken into consideration when making any dose adjustment.

##### Assay

Immunoassay is the most widely used commercial assay [99]. Currently there is no data indicating the superiority of any of the immunoassay methods over the others [100]. However, bias due to lack of between-method standardisation and high variability of measurement between methods is likely [101, 102]. Immunoassays remain appropriate for daily clinical TDM.

##### Dose adjustment strategies

Dose adjustments can be made by proportionally increasing or decreasing the dose relative to the ratio of the measured and the target concentration. The target concentrations commonly used for intermittent (15–20 mg/L) and continuous dosing (20–25 mg/L) are not the same with a higher continuous infusion target required to ensure the achievement of the same AUC as the intermittent dosing. Methodologies for dose individualization based on calculation of individual pharmacokinetic parameters and PK/PD targets (AUC/MIC) are available but not widely adopted in clinical practice [103]. Real time Bayesian forecasting coupled with TDM is thought to be most accurate for dose adaptation [104, 105].

No conclusive evidence supports the benefit of CI as a dose optimization strategy. It is not superior to intermittent dosing in terms of microbiological and clinical outcomes [106–111]. It may be considered though as a faster means to achieve consistent therapeutic concentrations given an adequate loading dose is used to avoid initial sub-therapeutic exposure [9, 112–114]. A recent meta- analysis [115] suggested a potential benefit of CI in reducing risk of nephrotoxicity.

#### Linezolid

The variability in linezolid pharmacokinetics was traditionally regarded less significant than with other antibiotics and consequently dose adjustments were considered unnecessary even in patients with renal and hepatic impairments [116]. However, accumulation of linezolid in renal insufficiency has been shown to be likely and results in toxicities such as pancytopenia, thrombocytopenia and liver dysfunction [117–120]. Reduced clearance has also been suggested in moderate hepatic insufficiency [121]. Contrasting reports exist on the possibility of disease related pharmacokinetic alterations. Consequently standard doses may result in a variable pharmacodynamic exposure [122], and are reported in the critically ill population with burns injuries [123, 124]. Elevated plasma concentration and associated risk of toxicity have also been reported [125–127]. In general, data to date indicates that TDM may be required in about 30 to 40% of patients to avoid dose-dependent toxicity as well as therapeutic failure [24, 122]. The impact of linezolid TDM on clinical outcome is yet to be demonstrated.

##### Selection of patients

A universal TDM program for linezolid is not supported based on current clinical data. Critically ill patients with sepsis, burns, pleural and peritoneal effusions, organ failure; patients infected with multidrug resistant bacteria; those receiving concomitant therapy with drugs that alter linezolid concentrations as well as those receiving long term linezolid therapy may benefit from TDM [121, 127, 128].

##### Sampling time

Trough concentrations are well correlated with AUC and are sufficient for linezolid TDM and estimation of an AUC/MIC ratio [122, 129]. The initial TDM sample should be taken just after pharmacokinetic steady state is achieved (usually considered on the third day of therapy).

##### Assay

HPLC methods have been published for linezolid TDM in plasma [128, 130], dried plasma spots [131, 132] and oral fluid [133] with good correlations between methods.

##### Dose adjustment strategies

Dose adjustments can be made by proportionally increasing or decreasing the dose in reference to the target concentration range (Table 3). CI may be a valuable strategy to provide a stable therapeutic exposure.

#### Fluoroquinolones

Difficult-to-predict pharmacokinetics of fluoroquinolones, particularly ciprofloxacin, can occur in critically ill patients as well as other patient groups. TDM may be beneficial given this pharmacokinetic variability to avoid treatment failure as well as minimise the emergence of resistance, particularly in the presence of less susceptible pathogens such as *Pseudomonas aeruginosa* which may have MICs of >0.5 mg/l [21, 134, 135].

Ciprofloxacin accumulation necessitating dose reduction has been reported in non-critically ill patients with renal impairment [136], although Van Zanten *et al.*[10] argued that dose reduction is unnecessary in critically ill patients despite their observation of increased AUCs. Other authors [137–139] also do not support dose reduction since accumulation is generally rare. It is likely that in patients with renal and gastrointestinal failure, dose reduction will be required as both clearance mechanisms will be affected. However, factors such as significant extracorporeal clearance due to RRT could influence variability of concentration in the critically ill [140, 141]. TDM may thus be an advantage for ciprofloxacin, although has yet to be described for levofloxacin or moxifloxacin.

##### Selection of patients

Universal TDM is not recommended and no specific patient groups have been shown to benefit most from TDM. Patients with infections caused by organisms with a high MIC (>0.5 mg/L) may benefit most, as traditional dosing is likely to result in sub-optimal exposure in high proportion of these patients.

##### Sampling time

At least two samples (peak and trough) should be taken to estimate the AUC. Both of these samples should be measured at steady state. The peak should be sampled in the post distribution phase, i.e. at least 30 min from the end of bolus infusion [137].

##### Assay

HPLC is the predominant method for measuring fluoroquinolones in plasma [142, 143] with dried blood spots [144] also used for TDM. A method using capillary electrophoresis has also been described [145] and immunoassay may be a more convenient future alternative [146].

##### Dose adjustment strategies

A wide range of targets has been proposed, however AUC/MIC of 125 or a C_max_/MIC of 8–10 is mostly accepted for treatment of Gram negative pathogens. A validated approach for dose adjustment is not currently available. Generally, to increase the AUC_0–24_, increasing the dose (e.g. IV 400 mg to 600 mg) or the frequency of dose (12-hourly to 8-hourly) are the more common methods for dose adaptation.

#### Daptomycin

TDM data on daptomycin is limited. The high protein binding and variable renal clearance make daptomycin a plausible candidate for TDM to increase the likelihood of achieving PK/PD targets [147–149]. TDM might also be useful in reducing the risk of musculoskeletal toxicity where it is highly associated with a trough concentration (C_min_) of >24.3 mg/L [150], especially when higher than standard doses are used. Current data is probably not sufficient to support a systematic TDM program for daptomycin. *In vivo* and small patient cohort studies reported efficacy cutoffs of AUC/MIC of 666 and C_max_/MIC of 59–94, the optimal PK/PD target for clinical application is yet to be elucidated [151, 152]. However, critically ill patients with sepsis, thermal burn injuries, profound hypoalbuminaemia, those infected by less susceptable bacteria, and those receiving RRT could potentially benefit from TDM-guided therapy as a means of ensuring achievement of PK/PD targets. Validated chomatographic assay methods are available for quantification of daptomycin [153], but given the high protein-binding of daptomycin and prevelance of hypoalbuminaemia in the critically ill, direct measurement of unbound drug concentrations might be preferred for clinical practice.

## Conclusion

TDM has traditionally served as a mechanism to minimize the toxicity of drugs. However, the approach to use TDM to maximize the therapeutic effects of less toxic compounds is becoming increasingly common. In the context of critical illness, there is strong data demonstrating that standard dosing regimens for many antibiotics frequently fail to provide optimal PK/PD exposure in critically ill patients. Given that pharmacokinetic exposures can be very difficult-to-predict in some patients, TDM is valuable to identify these patients and guide dose optimization. TDM can ensure attainment of PK/PD surrogate indicators of antibiotic efficacy, and therefore potentially improve patient outcome. A conservative approach to development of TDM programs is suggested because for many antibiotics, the personnel and resource costs are moderate and studies demonstrating conclusive clinical outcome advantages remain elusive. Based on the available data, a well-designed randomized controlled trial to determine the effect of TDM-guided dosing is supported.

## Abbreviations

AUC: Area under the concentration-time curve
*f* AUC/MIC: Ratio of area under the concentration–time curve of the unbound drug and the minimal inhibitory concentration of the pathogen
C_max_: Peak concentration during a dosing interval
C_min_: Trough concentration during a dosing interval
CLSI: Clinical and Laboratory Standards Institute
ECMO: Extracorporeal membrane oxygenation
EUCAST: European Committee on Antimicrobial Susceptibility Testing
HPLC: High-performance liquid chromatography
ICU: Intensive care unit
MIC: Minimal inhibitory concentration
PK/PD: Pharmacokinetics/pharmacodynamics
RRT: Renal replacement therapy
TDM: Therapeutic drug monitoring.
